# A Robust and Self-Paced BCI System Based on a Four Class SSVEP Paradigm: Algorithms and Protocols for a High-Transfer-Rate Direct Brain Communication

**DOI:** 10.1155/2009/864564

**Published:** 2009-04-28

**Authors:** Sergio Parini, Luca Maggi, Anna C. Turconi, Giuseppe Andreoni

**Affiliations:** ^1^Bioengineering Department, Politecnico di Milano University, 20133 Milan, Italy; ^2^INDACO Department, Politecnico di Milano University, 20133 Milan, Italy; ^3^IRCCS Eugenio Medea “La Nostra Famiglia”, 23842 Bosisio Parini, Lecco, Italy

## Abstract

In this paper, we present, with particular focus on the adopted processing and identification chain and protocol-related solutions, a whole self-paced brain-computer interface system based on a 4-class steady-state visual evoked potentials (SSVEPs) paradigm. The proposed system incorporates an automated spatial filtering technique centred on the common spatial patterns (CSPs) method, an autoscaled and effective signal features extraction which is used for providing an unsupervised biofeedback, and a robust self-paced classifier based on the discriminant analysis theory. The adopted operating protocol is structured in a screening, training, and testing phase aimed at collecting user-specific information regarding best stimulation frequencies, optimal sources identification, and overall system processing chain calibration in only a few minutes. The system, validated on 11 healthy/pathologic subjects, has proven to be reliable in terms of achievable communication speed (up to 70 bit/min) and very robust to false positive identifications.

## 1. Introduction

A brain-computer interface (BCI) is a system dedicated at providing its users with a new and alternative communication channel totally independent from the traditional output pathways of the nervous system such as peripheral nerves and muscles [1]. A BCI system achieves this goal by directly interfacing the cerebral activity, being it evoked or self-induced, with a common personal computer which nowadays represents a powerful and affordable platform for productivity, entertainment, worldwide communication, and remote control. Though strongly promoted and encouraged by military and entertainment research, its primal and main application is undoubtedly assistive technology for people affected by severe motor disabilities. In recent years, the research in the BCI field has grown rapidly, showing renewed interest and demonstrating how this communication system is in principle feasible. Nonetheless, present day solutions still have shortcomings that prevent their widespread application: limited information transfer-rate, high susceptibility to false positives, intense and wearing calibration sessions, and sensors intrusively are only a few of the major issues of current BCI systems.

In this paper we present, focusing on the adopted algorithmic and protocol-related solutions, a whole brain-computer interface system based on the steady-state visual evoked potentials (SSVEPs) paradigm. Stimulation at a certain frequency leads to oscillations at the same frequency and harmonics/subharmonics of the stimulation frequency [2]. In a BCI system, SSVEPs are used by simultaneously displaying several stimuli flickering at different frequencies. A user is able to select one specific stimulus by focusing on it consequently leading to an increased amplitude localized on those frequency bands related to the flickering frequency of the stimulus itself.

By considering other systems employing this particular BCI paradigm [2–5], the obtained bit-rates can be considered high when compared to other kinds of BCI systems (ranging from 30 to 68 bit/min depending on the protocol, the stimulation device, the number of classes, and the subject). In spite of the great advantage in terms of speed, SSVEP-based systems have been considered as “second class BCIs” over the years since a residual control of the gaze direction is needed (dependent BCIs). The lack of interest in SSVEP is reflected in the roughness of the current systems which implement basic mechanisms of adaptation to the user specific characteristics. Recent works [6] had renewed the interest in SSVEP-based systems: “*Many other important questions involving practical long-term use of SSVEP BCIs have not been addressed. Training with SSVEP BCIs can improve performance … the labels “dependent” and “independent” might be best regarded not as absolutes, but endpoints of a continuum.*” 

The main objectives of this study are guarantee a self-paced and asynchronous control maximizing communication performances in terms of attainable maximum bit-rate and robustness to false positives, provide a totally unsupervised biofeedback for an optimal mutual man-machine learning, and minimize the whole calibration phase duration.

## 2. Materials & Methods

The proposed SSVEP-based BCI system makes use of the BCI++ Framework [7, 8] as a technological platform for data acquisition, real-time algorithms management, protocols development, stimulation, and user interfacing. The framework structure can be summed up in two main modules able to communicate remotely by means of a TCP/IP socket based layer.

- *HIM Module*: the hardware interface module dedicated to the acquisition, storage, and visualization of the signal, the communication with the user interface, and the real-time execution of algorithms developed using C/C++ or Matlab environment;
- *AEnima Module*: the user interface module studied in order to simplify the implementation of new operating protocol for BCI-based user applications. This module was written using an OpenGL/Direct3D-based graphical engine in order to provide a more realistic and challenging experience to the user.

**Figure 1:**
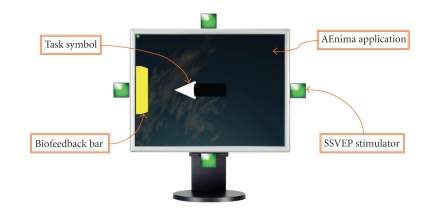
The stimulators placement and the graphic user interface layout.

The visual stimulation system consisted of four cubic spotlights with sides of 3 cm; each cube was mounted on a specific side of a standard 19” LCD monitor, thus allowing the user to ideally associate each light source to a 2-dimensional direction: up, down, left, or right (Figure 1). Each cube included a high-efficiency S-Flux green (*λ* = 500 nm) LED. In order to avoid direct exposition to the light and to diffuse the beam in a more efficient way, a matt film was put on the exposed face of the cube acting as a filter.

### 2.1. Operating Protocol

The adopted operating protocol aimed at gathering and confirming user-specific parameters for an optimal configuration of the processing and identification chain. The aim is that of overcoming potential intersubjects variability issues and guaranteeing the man-machine mutual-adaptation loop to start from the most reliable basis. The operating protocol consisted of the following sessions.

- Screening Session: to identify the most suitable stimulation frequencies for the subject.
- Training Session: to configure and train the processing and identification chain parameters.
- Testing Session: to validate and confirm the configuration parameters.

All the described sessions were guided by means of a specific graphic user interface developed using the AEnima module which also managed the stimulation device.

#### 2.1.1. The Screening Session

This session was structured in order to gather data useful for identifying the four most effective stimulation frequencies for the specific user: the amplitude of the SSVEP is not the same for different stimulation frequencies or different subjects [9]. The protocol consisted in two different phases:

- a stimulation phase: the user had to focus on a single light source for eight seconds;
- a resting phase: the stimulation was turned off, and the user had to focus on the center of the screen for eight seconds.

**Figure 2:**
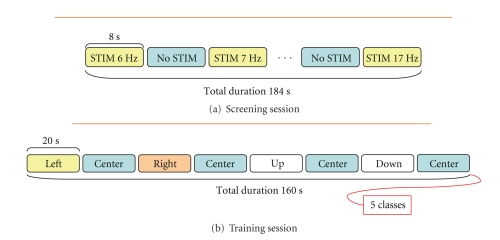
Timings and structures of the adopted (a) screening and (b) training protocols.

Each resting phase was followed by a stimulation phase at increased frequency resulting in a complete check of a predefined frequency range with a 1 Hz step (Figure 2(a)). The whole screening session lasted about 3 minutes (184 seconds).

According to [5], an optimal signal-to-noise ratio (SNR) of the steady-state visual evoked response can be usually achieved by stimulating in the 5–30 Hz range, with an SNR peak at 15 Hz which decreases at higher stimulation rate [9]. Moreover, according to [10], the late theta, the whole alpha band (6–13 Hz), and the early beta band (13–17 Hz) usually show better stimulus-related responses, while higher stimulation rate has proven to be reliable only when inducing a specific resonance phenomenon indicating a selective frequency preference of the neural oscillators of the subject. In this study, we decided to limit the inspected frequency range in the 6–17 Hz interval taking into account the following purposes:

- minimize the screening session duration;
- attain to the 5–30 Hz optimal range;
- include the theoretically optimal 15 Hz stimulation rate and its adjacent frequencies;
- minimize the inclusion of the mid-late beta (15–25 Hz) band because of its higher risk of inducing photoepileptic seizures [11];

A similar frequency range has been successfully adopted by other research groups [12]. In addition, the choice of stimulation frequencies in an SSVEP-based BCI application must ensure that the responses are as unique as possible. Thus, the stimulation frequencies were chosen in order to be neither harmonics nor subharmonics of each other [2].

**Figure 3:**
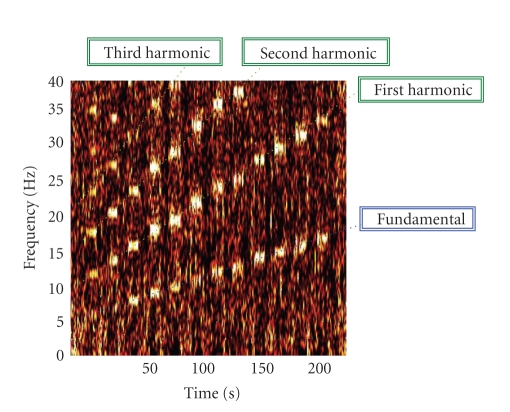
An example of a joint time-frequency analysis (JTFA) calculated on a screening dataset varying the stimulation frequency in the 6–17 Hz range.

The collected screening dataset was fed into a specific processing tool which calculated, for each common-average referenced electrode (each electrode referenced to the linked mastoids, see Figure 5), the joint time-frequency analysis (JTFA) by means of a short-time Fourier transform with a 4-seconds length (0.25 Hz frequency resolution) Kaiser window (*β* = 0.5) and 75% overlap. The JTFA parameters were chosen in order to guarantee a reasonable frequency resolution (0.25 Hz) limiting the time resolution (0.25 seconds) thus reducing computational demand. The choice of a Kaiser window was due to its desirable characteristics of minimizing both main lobe duration and side-lobes area thus reducing spectral leakage phenomena [13]. The JTFA results were plotted using a colormap-based graph and presented to the operator for visual inspection (Figure 3). Using the JTFA and according to the structure of the screening session (Figure 2), the operator could identify the response of each stimulation frequency (fundamental marker) and, consequently, the related response at higher or lower harmonics. For example, in the particular case presented in Figure 3, it is possible to notice a very weak fundamental response of the 6 and 7 Hz stimulations though showing a power increase of the harmonics of the stimulation rate. Higher stimulation frequencies clearly show both fundamental and harmonic response thus suggesting a more reliable visual evoked potential.

#### 2.1.2. The Training Session

This session aimed at collecting data useful for defining optimal processing and identification parameters for the specific user. During this session, all the four light sources were activated, and their flashing rates were set to the subject specific relevant stimulation frequencies identified at the end of the screening session. The protocol structure consisted in a guided sequence of stimulus gazing tasks; a symbol displayed on the LCD monitor guided the user to concentrate on a specific light source (left/right/up/down cue-symbol) or on the screen centre (dot-symbol) for 20 seconds following the sequence shown in Figure 2(b).

#### 2.1.3. The Testing Session

This session was used to verify and validate the performance of the system configured using the parameters defined in the previous sessions.

The protocol structure consisted in a guided sequence completion task. The user was asked to focus his/her attention on a particular light source, while the signal was processed and identified continuously by the self-paced online translation algorithm; the switching from one stimulus to another occurred only after the system identified a command related to the actual target source.

The sequence consisted of eight stimulus-specific tasks (two tasks per target) in random sequence: the classifier was activated four times per second, and a classification was considered reliable only when five consecutive and coherent classifications were produced. After each task, stimulation was temporarily stopped for three seconds before switching to the next task.

During this session, the unsupervised biofeedback was continuously provided to the user in order to help him/her in finding the best and most effective stimulus fixation strategy.

### 2.2. The Processing and Identification Chain

**Figure 4:**
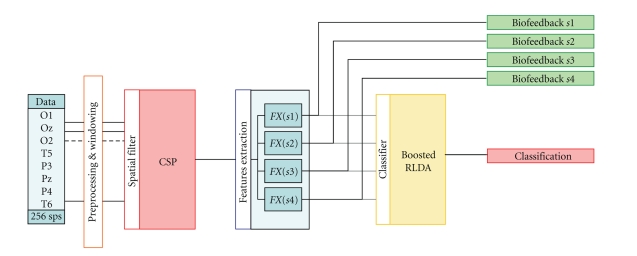
Conceptual scheme of the processing and identification chain for each of the four stimulation frequencies (*s*1, *s*2, *s*3, *s*4).

The processing and identification chain adopted for the real-time, self-paced translation algorithm is summarized in Figure 4. Each single block of the flow diagram is here described in details.

#### 2.2.1. Pre-Processing and Data Windowing

The 8-channel data acquired by the input device were continuously notch filtered on AC frequency using an elliptic filter with approximately 60 dB stopband attenuation. A further wide passband 5–45 Hz filter (4th-order FIR) was applied in order to reduce EOG/EMG artefacts. Preprocessed data were windowed using a 3-second length analysis window.

#### 2.2.2. Spatial Filtering and Channel Combining

Windowed data were spatially filtered using a single linear combiner with channel-specific weights. The aim of this processing block was that of optimizing the information contained in the 8-channel data in order to maximize the stimulus-related response signal-to-noise ratio with respect to basal activity.

Based on the assumption that an optimal spatial filtering was independent from the specific stimulation frequency, only a single linear combiner was adopted thus resulting in an 8-to-1 channel data compression. The spatial filter estimation was based on the training dataset and carried out using both a manual and an automatic approach.

The automatic approach was based on the common spatial patterns (CSPs) [14] method which has already proven to give reliable and effective results when used with other motor-imagery-based BCI applications [15–17]. Starting from two distributions in a C-dimensional space, being C the number of acquired channels, the CSP algorithm finds projections that maximize variance for one class while minimizing the variance of the other class. In the specific case of the SSVEP, the fixation of a light source flashing at a predefined frequency causes the brain to start “tuning” on that firing frequency thus producing a quasiperiodic response: by filtering the signal with a narrow bandpass filter centred on the stimulation frequency, this stimulus-related variation results in a higher variance signal with respect to the nonstimulus condition. From this point of view, the SSVEP signal seems well suited for the application of the CSP method intended as a technique for maximizing the SNR of the visual-evoked response against the nonstimulus condition.

According to the previously stated assumption, in order to exploit the information contained in the whole training dataset, the CSP estimation was applied on a repacked version of the training dataset consisting of 2 classes *stimulus* and *nonstimulus.* Each stimulus related portion of the dataset was narrow bandpass filtered (±0.2 Hz, stopband attenuation 30 dB) on the stimulation frequency and on its first and second harmonics. Similarly, each nonstimulus-related (NULL) portion was narrow bandpass filtered on the same frequency of the previous stimulation phase and on its harmonics. The contribution of each harmonics was considered additive. In this way, the 5 classes dataset (left, right, up, down, null) was repacked and consequently relabelled in a 2 classes dataset containing stimulus related and nonstimulus related trials. Calling *X* the repacked dataset, let Ω_S_ and Ω_NS_ be, respectively, the pooled estimates of the covariance matrices for the *stimulus* and *nonstimulus* related classes, as follows:

(1) $$\Omega_{\text{class}}=\frac{1}{N_{\text{class}}}\overset{N_{\text{class}}}{\underset{i=1}{\sum }}\;X_{i}X_{i}^{T}\;\text{with}\;\;(\text{class}\in \{\text{S},\text{NS}\}),$$

where *N*_class_ is the number of trials for class, and *X*_*i*_ is the matrix (in the form (*channels*, *sample*)) containing the portion of data corresponding to the *i*th trial. The two covariance matrices, Ω_S_ and Ω_NS_, are simultaneously diagonalized such that the eigenvalues sum to one. This is achieved by calculating the generalized eigenvectors *V* as follows:

(2) $$\Omega_{\text{S}}V=(\Omega_{\text{S}}+\Omega_{\text{NS}})VL,$$

where *L* is the diagonal matrix containing the generalized eigenvalues, and the column vectors of *V* are the filters for the CSP projections. Theoretically, in the specific case of SSVEP, the best contrast is provided only by those eigenvectors corresponding to eigenvalues close to 1 (large variance for class S and small variance for class NS). Thus, in this study, we chose to consider only the filter corresponding to the highest eigenvalue. According to [15], useful information is also contained by those projections corresponding to eigenvalues close to the highest; in this specific work, we decided not to consider this information in order to minimize the feature vector dimensionality and maximize classifier robustness with small training dataset.

The manual approach relied on a software tool developed for the selection of channels combinations. The operator was asked to specify the type of contribution related to each electrode: *positive-contribution, negative-contribution*, and *null-contribution*. The resulting data channel was calculated by subtracting the mean of the *negative-contribution* electrodes from the mean of the *positive-contribution* electrodes, ignoring the *null-contribution* channels. The manual selection of the channels combination was aided by providing the operator with a binary class separability index (Section 2.2.3) calculated, for each stimulation frequency, using the features extracted from the idle portion and the frequency-specific stimulation portion of the training dataset. This specific approach was considered in order to give the operator the possibility to evaluate, aided by the CSP mapping, the exclusion of channels showing low discriminative power (filter weight close to 0), thus optimizing the subject specific electrodes montage.

#### 2.2.3. Features Extraction and Evaluation

The signal feature adopted in this study is an adaptation of the stimulus-locked average frequently used for the extraction of evoked potential. In this case, no flash-locked triggering was used but instead we assumed that each event-related response has duration equal to the specific *s*th stimulation period *p*_*s*_. According to this, the windowed and spatially combined signal *X*_win_ = [*x*_1_, *x*_2_,…, *x*_*N*_] was divided into a number *N*_SW_ of subwindows of length *p*_*s*_ and averaged synchronously, thus

(3) $${\text{B}}_{s}=[\beta_{s}(1),\beta_{s}(2),\ldots ,\beta_{s}(p_{s})],$$

where

(4) βs(i)=1NSW⋅∑j=1j=NSW Xwin(i+(j−1)⋅ps),being   NSW=Nps,

where the signal components, synchronous with the stimulation frequency, were consequently enhanced while the other components were reduced.

An amplitude estimation of the SSVEP was obtained by means of the following equation:

(5) $$FX(s)=\frac{\sigma ({\text{B}}_{s})}{\sigma (X_{\text{win}})},$$

where *σ*(B_*s*_) is the standard deviation of the average (time-locked to the *s*th stimulus), *σ*(*X*_win_) is the standard deviation calculated using the whole data window, and *FX*(*s*) is the resulting normalized feature, estimation of the response to the *s*th stimulus. The resulting feature is a numerical value which theoretically ranges from zero, in the case of total disruptive interference, to one, when the signal has only one periodic component with the same period of the stimulation (constructive interference). The main advantage of this property is that the resulting values do not need to be scaled on the basis of a previous training session thus allowing the operator to provide the user with a consistent biofeedback right after the initial screening session. Only one feature per stimulus was extracted thus resulting in a final features vector of 4 elements.

Once the features were extracted, an estimation of the discriminative power of the system was provided in the form of a Bhattacharyya distance calculated between each stimulation-related portion of the features-set and its consequent nonstimulation portion according to the training dataset structure (Figure 2(b)).

The Bhattacharyya bound is a special case of the Chernoff bound [16, 17]. Often, the Bhattacharyya bound is used instead of the Chernoff bound since empirical evidence indicates it to be optimal when the majority of the separation comes from a difference in classes means [18]. Recently, it has also been shown that it is feasible to predict the classification error accurately using the Bhattacharyya distance [19, 20]. The expression for the Bhattacharyya distance is

(6) $$\begin{array}{ll} \overset{¯}{\mu }(c) & =\frac{1}{8}{\left({\overset{¯}{M}}_{\text{NS}}-{\overset{¯}{M}}_{c}\right)}^{T}{\left[\frac{{\overset{¯}{S}}_{c}+{\overset{¯}{S}}_{\text{NS}}}{2}\right]}^{-1}({\overset{¯}{M}}_{\text{NS}}-{\overset{¯}{M}}_{c})\\ & \;+\frac{1}{2}\ln\frac{\left[({\overset{¯}{S}}_{c}+{\overset{¯}{S}}_{\text{NS}})/2\right]}{\sqrt{\left|{\overset{¯}{S}}_{c}\right|\left|{\overset{¯}{S}}_{\text{NS}}\right|}}\\ & \;\;\;\;\text{with}\;\;(c\in \{\text{left},\text{right},\text{up},\text{down}\}), \end{array}$$

where ${\overset{¯}{M}}_{c}$ and ${\overset{¯}{S}}_{c}$ are, respectively, the vectors of means and variances of the class *c* of the feature-set, while ${\overset{¯}{M}}_{\text{NS}}$ and ${\overset{¯}{S}}_{\text{NS}}$ are, respectively, the vectors of means and variances of the nonstimulus class of the feature-set. $\bar{\mu }(c)$ is a vector of 4 elements containing the discriminative power of each feature with respect to class *c*. An effective configuration of the system would present, for each class *c*, a high discriminative power of the features related to the class specific stimulus while indeces close to zero for the other features.

#### 2.2.4. The Classifier

The previously described features set was used to train a 5-class (left, right, up, down, null) supervised classifier based on the Regularized Linear Discriminant Analysis method [18, 21]. Linear Discriminant Analysis (LDA) is a well-known classification method that projects high-dimensional data onto a low-dimensional space where the data are reshaped to maximize class separability. The optimal projection or transformation in classical LDA is obtained by minimizing the within-class distance and maximizing the between-class distance simultaneously, thus achieving maximum discrimination.

In this study, we adopted a regularized version of the discriminant analysis method using a double parameter (*λ*, *γ*) model selection scheme as described in [21]. The proposed identification algorithm also included a boosting technique based on the Freund and Schapire method as described in [22]; classifiers are constructed on weighted versions of the training set, which are dependent on previous classification results. Initially, all objects have equal weights, and the first classifier is constructed on this dataset. Then, weights are changed according to the performance of the classifier. Erroneously classified objects get larger weights, and the next classifier is boosted on the reweighted training set. In this way, a sequence of classifiers is obtained, which are then combined by a weighted majority vote in the final decision.

## 3. Results

The system was tested on two different control groups made of volunteers without previous BCI experiences. The first control group (G1) was composed of 7 healthy subjects, aged from 18 to 50 years. The second control group (G2) was composed of 4 pathological subjects, aged from 10 to 30, and affected by Duchenne muscular dystrophy at different stages. Every subject suffering from defects of vision performed the whole experimental session wearing appropriate corrective lens. During acquisitions, the subject sat on a comfortable chair at a distance of about 80 cm from an LCD monitor in a noise-controlled and slightly dim room.

**Figure 5:**
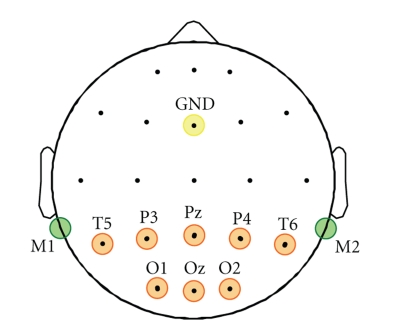
The adopted electrodes montage includes orange highlighted channels {T5,P3,Pz,P4,T6,O1,Oz,O2} using a linked mastoids reference and a ground channel placed in GND position.

The EEG signal was acquired at 256 samples per second by means of an 8-channel wearable system [23] developed by Sensibilab and WoWS!-Lab (Politecnico di Milano University, Italy): the communication with the HIM Module was achieved through a Bluetooth (BT) wireless connection. In this study, we used a g.EEGcap (g.Tec GmbH, AU, http://www.gtec.at/) with a set of eight gold—plated electrodes placed over the occipital area {T5,P3,Pz,P4,T5,O1,Oz,O2} with a linked-mastoids reference as illustrated in Figure 5.

The adopted experimental protocol was the same for both control groups and, according to the previously described protocol structures, it consisted in the following:

- a screening session (duration: 184 seconds);
- a training session (duration: 160 seconds);
- a testing session (duration: depending on user's performances).

In Table 1, we report, for each subject of group G1 and G2, the global misclassification rate, the typical delay time, and the resulting bit-rate obtained using the CSP-based spatial filtering approach. Though each subject performed the whole experimental session using a system configured on a 3-second long analysis window, results obtained by simulating the testing session using an analysis window of, respectively, 2 and 4 seconds length are also presented.

The misclassification rate was calculated taking into account the self-paced nature of the identification chain, thus increasing the errors count every time the system identified a control state (left, right, up, down) different from the actual control task. The typical delay time consisted in the mean time interval between the control task trigger and the first correct identification.

The bit-rate was calculated according to the popular definition proposed by Wolpaw and based on Shannon's definition of information rate for noisy channels [24] as follows:

(7) BR=V⋅R,

where

(8) $$R=\log_{2}N+P\cdot \log_{2}P+(1-P)\cdot \log_{2}\frac{1-P}{N-1},$$

where BR is the bit-rate in bit/min, *V* is the classification speed in symbols/min, and *R* is the information carried by one symbol in bit/symbol. The information carrier *R* was calculated considering *N* as the number of actual control states (*N* = 4) and *P* as one minus the global error rate. This definition has widely been used by other research groups (see [24]) in order to evaluate and quantify BCI communication performances, and it is consequently adopted in this study for the sake of comparability.

The error rate ranges from 0 to approximately 7%. Considering a window length of 3 and 4 seconds, the 80% of the analyzed subjects obtained an error rate comprised from 0 to 1% showing an almost perfect robustness of the system to false positives. The use of a window length of 2 seconds increases the error rate yet showing an average across subjects of about 2.5% which, together with the reduction of the typical delay time, reflects the dramatic gain in terms of bit-rate achieved by the system using small data windows. For every subject, with the only exception of subject S13, the bit-rate ranges from 35 to 70 bit/min with an average across subjects of 51.47 bit/min, a minimum of 17 bit/min, and a maximum of almost 70 bit/min. All the subjects completed the whole testing protocol in less than 80 seconds. The minimum duration of the testing session across all subjects was achieved by subject S12 with an actual session duration (including the pause of three seconds after each task, Section 2.1.3 of approximately 50.85 seconds.

**Figure 6:**
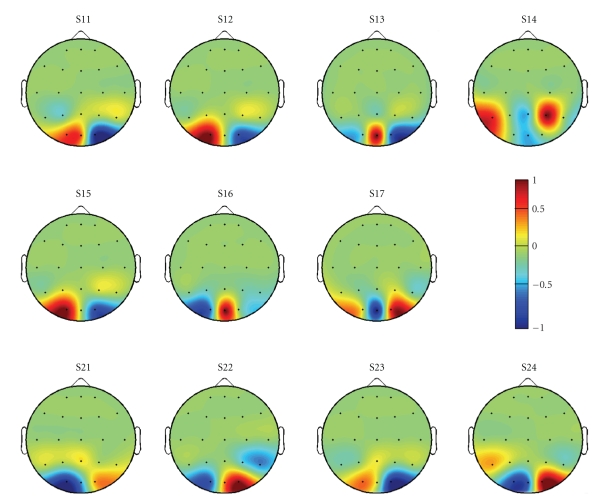
Normalized weights associated to each electrode of the considered electrodes montage calculated using the Common Spatial Patterns method. Most of the subjects show a dominant activation localized over the O1-Oz-O2 electrode positions and, according to the weight signs, the optimal filter is usually a bipolar derivation possibly reinforced by parietotemporal activations.

All the results are calculated using the CSP-based spatial filtering approach. In Figure 6, we report, for each subject of group G1 and G2, the normalized weight associated to each electrode of the considered montage. Most of the subjects show a dominant activation localized over the O1-Oz-O2 electrode positions and, according to the weight signs, the optimal filter is usually a bipolar derivation (e.g., O1-O2 for S15 and O1-Oz for S16) possibly reinforced by parietotemporal activations (e.g., subject S24). Such distributions suggest that a subject specific optimization of the electrodes montage could provide interesting results using only few electrodes, thus a future investigation in this terms is mandatory.

**Figure 7:**
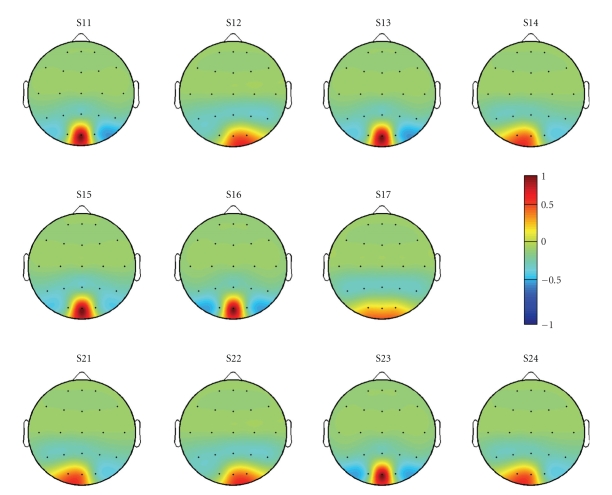
Weights associated to each electrode of the considered electrodes montage using the manual channels combining approach described in Section 2.2.2. Positive weights refer to *positive-contribution* electrodes, while negative weights refer to *negative-contribution* electrodes.

For sake of completeness, in Table 2 we also provide results obtained by means of the standard manual channel combining approach. By comparing the bit-rates obtained using the manual and the CSP method for each subject and for each length of the analysis window, the advantages of the proposed spatial filtering method are clear: the CSP method allows for a considerably lower error rate without worsening the delay time of the SSVEP response identification, thus guaranteeing a huge increase in the achievable information transfer rate. In Figure 7, we also report, for each subject, the normalized weight associated with each electrode of the considered montage using the manual combining approach described in Section 2.2.2.

**Figure 8:**
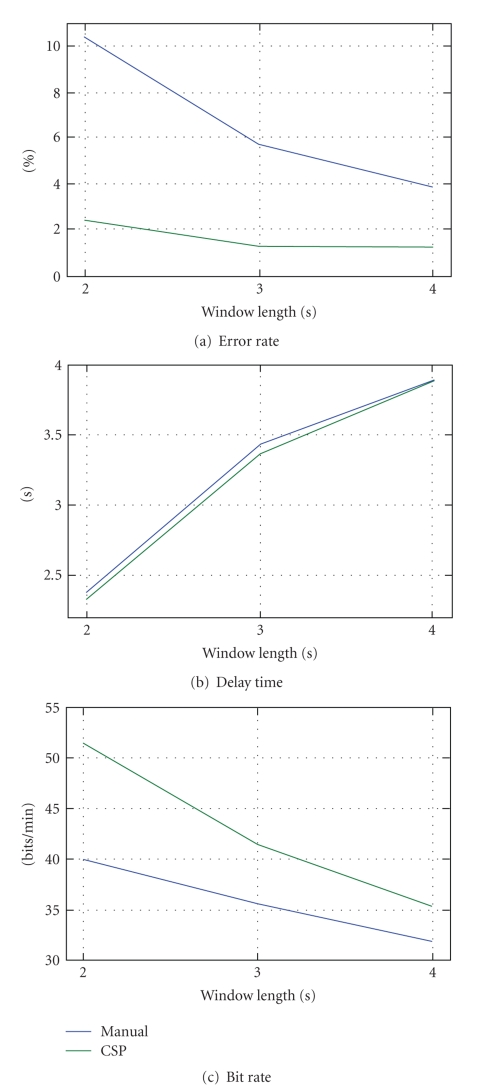
Average results across all subjects (G1 + G2) obtained using the manual (blue line) and CSP-based (green line) methods with different lengths of the analysis window; (a) error rate, (b) delay time, and (c) bit-rate calculated using Wolpaw's definition.

In Figure 8, for each spatial combining method, we provide (a) the average error rate, (b) delay time, and (c) bit-rate calculated across all the subjects from both control groups (G1 and G2) using different lengths of the data analysis window (2, 3, and 4 seconds). It is possible to notice how the adoption of the CSP method dramatically reduces the error rate yet retaining a comparable delay time of the SSVEP response when compared to the manual channel selection approach. The gap in the resulting error rate is thus reflected in the final bit-rate estimation. In Figure 8(a), the error rate obtained using the CSP method is almost constant between each considered window length. Consequently, the bit-rate gap progressively reduces by increasing the data analysis window length; the proposed CSP-based method is a valuable solution for increasing the identification robustness using small data portion thus offering faster response time and higher information transfer-rates.

**Figure 9:**
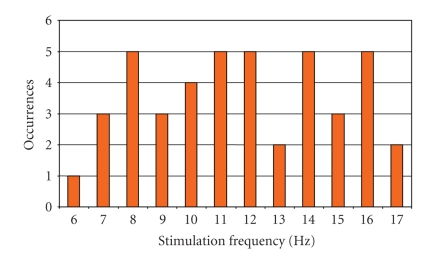
Occurrences of each stimulation frequency of the screened 6–17 Hz range calculated on both G1 and G2 control groups.

In Figure 9, a histogram calculated on the basis of the occurrences of each stimulation frequency in the predefined range (6–17 Hz) is presented. Though beyond the scope of our work, we could notice that, in spite of a low response to the 6 Hz stimulation, no clearly predominant stimulation pattern can be inferred from the results obtained on the considered control groups. This could be seen as a further confirmation of the importance of the identification of subject-specific relevant stimulation frequencies. 

## 4. Discussion and Conclusion

In this work, novel methods dedicated to the SSVEP paradigm are proposed in order to guarantee user specific customization and increase man-machine interaction. Figure 9 supports the introduction of a method for the identification of the subject specific frequencies. The proposed method requires a session of only 184 seconds. The analysis of the SSVEP response using the JTFA analysis is very intuitive.

As shown in Figure 6, there is predominance in the spatial distribution of the SSVEP response but at least half of the CSP distributions differ from this standard. As shown in Tables 1 and 2, the proposed user specific adaptation significantly increases the performance for subject with an average SSVEP response amplitude and also gives an operator error proof method when compared to the manual method (Figure 7). The application of the Common Spatial Patterns method to the SSVEP paradigm has proven to be reliable and to provide an effective mapping of the relevant signal. This method provides a fast and totally automated solution to the spatial filtering problem and represents a good starting point for electrodes montage optimization: aided by the proposed features ranking index (Bhattacharyya distance), the operator should be able to exclude from the user-specific montage those channels showing a spatial weight close to zero thus minimizing user's setup time for future control sessions.

The adopted signal feature demonstrated to be effective in the discrimination of the different control tasks retaining a high discriminative power; the theoretical strength of such a feature resides in its ability to take into account an increase of energy both in the fundamental and harmonic frequencies of the stimulation. The autoscaling property is crucial when used for providing the user with a biofeedback independent from the calibration of the classification module; in this way we propose an alternative way to solve the common *man-machine learning dilemma* (MMLD) already discussed in [25] and stating that two systems (man and machine) are strongly interdependent but have to be adapted independently in order to obtain a well-balanced mutual adaptation.

The goal of maximizing the communication performances was reached by guaranteeing an average bit-rate of approximately 51.5 bit/min with a transfer rate peak at about 70 bit/min for more than one tested subject. The overall system robustness with respect to false positives was achieved by means of an average error rate across subjects of about 2.5% even using a data analysis window of 2-second length. The three-second length analysis window has proven to be a reasonable trade-off between maximization of the information transfer-rate and minimization of the error though, with many subjects, the use of a 2-second window led to a dramatic gain in communication performances yet showing an error rate almost close to zero. Regarding this, it is also important to point out that every tested subject was facing for his/her very first time a BCI-based communication system and that each user performed the whole experimental session only once.

Maintaining that the dimensionality of the G1 and G2 control groups prevents any robust statistical analysis (which is also beyond the scope of this work); there is no evidence of performance differences between healthy and pathological subjects.

Future works will include the evaluation of the performance of the system with manually and automatically optimized electrodes montage and investigations of the attainable control using user-specific stimulation frequency in the gamma range (>30 Hz) which could theoretically lead to a less annoying and safer stimulation thanks to vision persistence.

## Figures and Tables

**Table 1:** Results using the CSP method for automatic spatial filtering as described in Section 2.2.2. Error rate, typical delay time, and bit-rate calculated on the basis of the testing session performed by each subject of group G1 and G2 using different data analysis window lengths. For each group, the average values across subject are presented (MEAN). A global results calculated on every subject from both control groups is also provided (G1 + G2).

	ID	Data window length								
		2 seconds			3 seconds			4 seconds		
		ERR [%]	DLY [sec]	**BR** [bit/min]	ERR [%]	DLY [sec]	**BR** [bit/min]	ERR [%]	DLY [sec]	**BR** [bit/min]
G1	S11	0.57	2.25	**51.83**	0.00	2.84	**42.26**	0.00	3.29	**36.53**
	S12	0.62	1.68	**69.31**	0.00	2.30	**52.16**	0.62	2.58	**44.98**
	S13	7.03	5.39	**16.93**	6.83	11.33	**8.11**	6.21	11.93	**7.88**
	S14	0.00	3.25	**36.88**	0.00	3.63	**33.07**	0.00	3.97	**30.21**
	S15	3.01	2.49	**42.37**	0.00	3.18	**37.79**	0.00	3.66	**32.79**
	S16	2.26	1.77	**61.32**	0.00	2.33	**51.46**	0.00	2.85	**42.14**
	S17	4.97	1.71	**57.24**	4.42	2.46	**40.62**	2.76	3.02	**35.25**
	**MEAN**	2.64	2.65	**47.98**	1.61	4.01	**37.92**	1.37	4.47	**32.82**

G2	S21	0.00	1.77	**67.81**	0.00	2.18	**55.15**	0.00	2.55	**47.04**
	S22	5.42	2.05	**47.10**	3.01	2.71	**38.95**	4.82	3.21	**30.78**
	S23	1.18	1.89	**59.81**	0.59	2.49	**46.74**	0.00	2.94	**40.80**
	S24	2.40	1.94	**55.60**	0.00	2.38	**50.44**	0.00	2.93	**41.01**
	**MEAN**	2.25	1.91	**57.58**	0.90	2.44	**47.82**	1.20	2.91	**39.91**

	**G1 + G2**	2.50	2.38	**51.47**	1.35	3.44	**41.52**	1.31	3.90	**35.40**

**Table 2:** Results using the manual channels combining approach described in Section 2.2.2. Error rate, typical delay time, and bit-rate calculated on the basis of the testing session performed by each subject of group G1 and G2 using different data analysis window lengths. For each group, the average values across subject are presented (MEAN). A global results calculated on every subject from both control groups is also provided (G1 + G2).

	ID	Data window length								
		2 seconds			3 seconds			4 seconds		
		ERR [%]	DLY [sec]	**BR** [bit/min]	ERR [%]	DLY [sec]	**BR** [bit/min]	ERR [%]	DLY [sec]	**BR** [bit/min]
G1	S11	17.05	1.62	**39.64**	9.66	2.81	**29.67**	5.11	3.28	**29.80**
	S12	0.00	1.99	**60.35**	0.00	2.27	**52.87**	0.62	2.71	**42.90**
	S13	14.34	5.83	**12.14**	10.83	11.21	**7.14**	10.39	11.77	**6.90**
	S14	0.00	2.75	**43.57**	0.00	3.44	**34.87**	0.00	3.96	**30.33**
	S15	3.61	2.04	**50.66**	1.20	2.57	**44.11**	0.00	3.14	**38.16**
	S16	10.73	1.74	**46.18**	2.26	2.55	**42.54**	0.56	3.24	**35.96**
	S17	25.97	1.62	**28.21**	13.81	2.25	**32.10**	8.84	2.68	**31.95**
	**MEAN**	10.24	2.51	**40.11**	5.39	3.87	**34.76**	3.65	4.40	**30.86**

G2	S21	4,80	1,90	**51,98**	4,10	2,22	**45,62**	3,12	2,80	**37,50**
	S22	12,30	2,20	**34,55**	7,12	2,81	**32,38**	5,50	3,25	**29,64**
	S23	10,06	2,24	**36,72**	6,51	2,58	**36,01**	7,10	3,02	**30,16**
	S24	16,17	1,82	**36,52**	8,38	2,47	**35,22**	1,80	2,94	**37,57**
	**MEAN**	10,83	2,04	**39,94**	6,53	2,52	**37,31**	4,38	3,00	**33,72**

	**G1 + G2**	10.45	2.33	**40.04**	5.80	3.37	**35.68**	3.91	3.89	**31.90**

## References

[1] Wolpaw JR, Birbaumer N, McFarland DJ, Pfurtscheller G, Vaughan TM (2002). Brain-computer interfaces for communication and control. *Clinical Neurophysiology*.

[2] Müller-Putz GR, Scherer R, Brauneis C, Pfurtscheller G (2005). Steady-state visual evoked potential (SSVEP)-based communication: impact of harmonic frequency components. *Journal of Neural Engineering*.

[3] Gao X, Xu D, Cheng M, Gao S (2003). A BCI-based environmental controller for the motion-disabled. *IEEE Transactions on Neural Systems and Rehabilitation Engineering*.

[4] Lalor EC, Kelly SP, Finucane C (2005). Steady-state VEP-based brain-computer interface control in an immersive 3D gaming environment. *EURASIP Journal on Applied Signal Processing*.

[5] Middendorf M, McMillan G, Calhoun G, Jones KS (2000). Brain-computer interfaces based on the steady-state visual-evoked response. *IEEE Transactions on Rehabilitation Engineering*.

[6] Allison BZ, McFarland DJ, Schalk G, Zheng SD, Jackson MM, Wolpaw JR (2008). Towards an independent brain-computer interface using steady state visual evoked potentials. *Clinical Neurophysiology*.

[7] Maggi L, Parini S, Perego P, Andreoni G BCI++: an object-oriented BCI prototyping framework.

[8] Perego P, Maggi L, Parini S, Andreoni G A home automation interface for BCI application validated with SSVEP protocol.

[9] Pastor MA, Artieda J, Arbizu J, Valencia M, Masdeu JC (2003). Human cerebral activation during steady-state visual-evoked responses. *The Journal of Neuroscience*.

[10] Herrmann CS (2001). Human EEG responses to 1–100 Hz flicker: resonance phenomena in visual cortex and their potential correlation to cognitive phenomena. *Experimental Brain Research*.

[11] Fisher RS, Harding G, Erba G, Barkley GL, Wilkins A (2005). Photic- and pattern-induced seizures: a review for the epilepsy foundation of america working group. *Epilepsia*.

[12] Martinez P, Bakardjian H, Cichocki A (2007). Fully online multicommand brain-computer interface with visual neurofeedback using SSVEP paradigm. *Computational Intelligence and Neuroscience*.

[13] Proakis JG, Manolakis DG (1996). *Digital Signal Processing: Principles, Algorithms and Applications*.

[14] Koles ZJ (1991). The quantitative extraction and topographic mapping of the abnormal components in the clinical EEG. *Electroencephalography and Clinical Neurophysiology*.

[15] Ramoser H, Müller-Gerking J, Pfurtscheller G (2000). Optimal spatial filtering of single trial EEG during imagined hand movement. *IEEE Transactions on Rehabilitation Engineering*.

[16] Dornhege G, Blankertz B, Krauledat M, Losch F, Curio G, Müller K-R (2006). Optimizing spatio-temporal filters for improving brain-computer interfacing. *Advances in Neural Information Processing Systems*.

[17] Blankertz B, Tomioka R, Lemm S, Kawanabe M, Müller K-R (2008). Optimizing spatial filters for robust EEG single-trial analysis. *IEEE Signal Processing Magazine*.

[18] Fukunaga K (1990). *Introduction to Statistical Pattern Recognition*.

[19] Choi E, Lee C (2003). Feature extraction based on the Bhattacharyya distance. *Pattern Recognition*.

[20] Lee C, Choi E (2000). Bayes error evaluation of the Gaussian ML classifier. *IEEE Transactions on Geoscience and Remote Sensing*.

[21] Friedman JH (1989). Regularized discriminant analysis. *Journal of the American Statistical Association*.

[22] Freund Y, Schapire RE Experiments with a new boosting algorithm.

[23] Maggi L, Piccini L, Parini S, Andreoni G, Panfili G Biosignal acquisition device—a novel topology for wearable signal acquisition devices.

[24] Kronegg J, Voloshynovskiy S, Pun T Analysis of bit-rate definitions for brain-computer interfaces.

[25] Pfurtscheller G, Neuper C (2001). Motor imagery direct communication. *Proceedings of the IEEE*.

